# Cold Binary Atomic Collisions in a Light Field

**DOI:** 10.6028/jres.101.050

**Published:** 1996

**Authors:** Paul S. Julienne

**Affiliations:** National Institute of Standards and Technology, Gaithersburg, MD 20899-0001

**Keywords:** binary atomic collision, Bose-Einstein Condensation, cold trapped atoms, Franck-Condon factor, photoassociation spectrum, spectral line shape

## Abstract

The rate coefficients are calculated for trap loss due to excited state formation during *s*-wave collisions of two atoms in a light field in a cold atomic gas near conditions for formation of a Bose-Einstein condensate. Blue detuning from the allowed atomic resonance transition causes excitation of a replusive molecular potential, whereas red detuning causes excitation when the light is tuned near a bound vibrational energy level of an attractive molecular potential. In either case, when the light intensity is low and the detuning is large compared to the natural linewidth of the atomic transition, the rate coefficient for the collisional loss rate is proportional to a molecular Franck-Condon factor. A simple reflection approximation formula is derived which permits the rate coefficient to be given analytically in either case. The Franck-Condon factor is equal to |*Ψ*_g_(*R*_C_)^2^|/*D*_C_, where *Ψ*_g_(*R*_C_) is the ground state scattering wavefunction at the Condon point *R*_C_, where the quasimolecule is in resonance with the exciting light, and *D*_C_ is the slope difference between ground and excited potentials at *R*_C_. The analytic reflection approximation formula, as well as a simple phase-amplitude formula for the intermediate range wavefunction, give excellent agreement with the results of numerical quantum mechanical calculations. The trap loss rates due to binary collisions are comparable to or exceed those due to atomic recoil heating for a wide range of detunings to the blue of atomic resonance and near the peaks of photoassociation resonances for the case of red detuning.

## 1. Introduction

Atomic collisions play a crucial role in the phenomena of Bose-Einstein condensation (BEC) [1–3]. Elastic momentum-transfer collisions control the rate of evaporative cooling leading to the high phase-space density required for the formation of a condensate. The ground state scattering length, also a property of elastic collisions, controls the nonlinear coupling parameter in the nonlinear Schrodinger equation for the condensate wavefunction [4]. Inelastic collisions among condensate atoms also determine a lower bound to the loss rate of the condensate (other processes may contribute to loss as well) and thereby help determine the condensate life-time. Therefore, it is crucial to understand these ground state collisions. Calculating their rates requires that we be able to compute the ground state scattering wavefunction. Although these collisions occur in the absence of light, photoassociation spectroscopy offers a powerful probe of the ground state wavefunction [5–8]. Light also has the prospect for being an important probe of condensate properties and for manipulating a condensate or even the collisions within a condensate. Therefore, it is crucially important to understand how light affects the atoms in a cold, dense atomic gas near the BEC regime.

This article will be concerned with understanding the rates of binary collisions in a weak, off-resonant light field in a cold atomic gas. Since only the case of a weak radiation field is treated, the effect of light on the binary collision can be described perturbatively. The case of a strong saturating field will be treated separately [9]. The detuning is assumed to be large enough compared to the natural atomic linewidth *γ*_A_ and the density *N* moderate enough that binary events are sufficiently isolated that three-body collisions can be ignored, as well as collective effects [10]. Certainly, considering binary events is the first step in understanding the effect of light on interacting atoms. The object is to provide a simple framework with which to understand these binary events. They would need to be incorporated at a later stage into a more comprehensive understanding of the dynamics and stability of an atomic gas near BEC. Elsewhere, we give a brief account of the possibility of using photoassociation spectroscopy to probe many-body interactions in a condensate [11].

Another important reason for having a simple model for understanding these binary collisions in a light field is to understand how spectroscopic methods can be used as an experimental tool for probing the ground state scattering wavefunction. In particular, we have proposed that high resolution photoassociation spectroscopy would provide an especially sensitive probe of the ground state potential and scattering properties [5]. In fact, this technique has been used to place bounds on scattering lengths for Li [8], Rb [6], and Na [12, 13] ground state collisions. Even tighter bounds should be possible with improved experiments. In particular, we will show below how the spectroscopy of very cold gases might be use to measure properties of the ground state wavefunction.

The primary effect of light on cold trapped atoms is to cause loss processes by giving the atoms enough energy to escape the trap. For example, a single atom scatters light of frequency *ω* detuned ***Δ***_A_ = *ω* − *ω*_A_ from the atomic resonance frequency *ω*_A_ at the rate,

$$\gamma_{\text{atomic}}=\gamma_{A}{\left(\frac{\Omega_{A}}{\Delta_{A}}\right)}^{2},$$ (1)

where $\Omega_{A}=\sqrt{2\pi I/c}$ *d*_A_ is the Rabi frequency at laser intensity *I* for the atomic transition with transition dipole *d*_A_, and we assume |***Δ***_A_| ≫ *Ω*_A_ and |***Δ***_A_| ≫ *γ*_A_; ***Δ***_A_ is defined so as to be positive for blue detuning and negative for red. Since each scattering event gives a recoil momentum kick of *ℏk_ω_* = *ℏω*/*c* to the atom, and since the thermal momentum of the atom is comparable to, or smaller than, *ℏk_ω_*, this atomic light scattering heats the atoms, causing their loss from the trap.

**Fig. 1:**
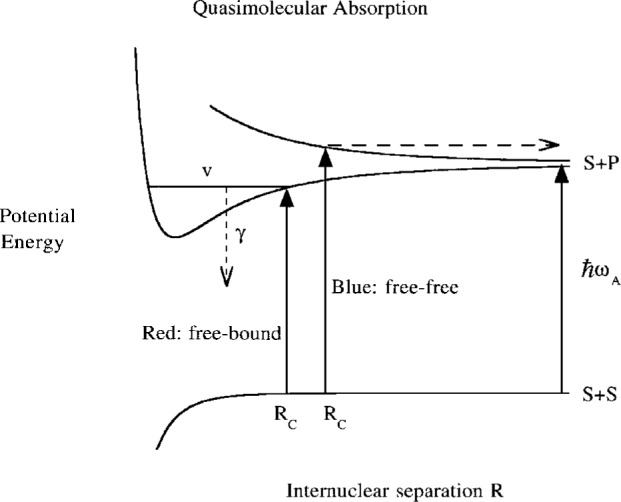
Schematic diagram of quasimolecular potential energy curves for two atoms with an allowed *S* → *P* transition, showing free-bound and free-free absorption for respective red and blue detuning from the atomic resonance frequency *ω*_A_.

Binary events also cause loss of atoms from a trap, but with a rate which is very different for the cases of red and blue detuning. Figure 1 illustrates the two cases schematically, using the molecular potential energy curves for the quasimolecule comprised of the two colliding atoms. Blue detuning excites the ground state atoms to a repulsive molecular state, causing the production of excited atoms with kinetic energy *E*_e_ = *E*_g_ + *ℏ****Δ***_A_. This heating has been discussed in the context of optical shielding [14,15,9]. The probability for this free-free process is a smooth function of detuning. On the other hand, red detuning excites the ground state atoms to a bound eigenstate of the attractive molecular state. This only happens with high probability if the light is tuned close to exact resonance with the energy *E*_υ_ of the bound vibrational state *υ*. Since the decay of the bound state mostly leads to products that are not trapped, this process gives rise to a trap-loss photoassociation spectrum, which is now well studied for the alkali dimers [16–21]. The probability for this free-bound process is a sharply peaked function of detuning. We have every reason to expect that a gas near BEC conditions, or even a condensate itself, will possess a detailed photoassociation spectrum for detuning on the red side of *ω*_A_.

When the laser intensity *I* is sufficiently low, the probability for both red and blue detuning loss processes can be described by a Franck-Condon factor between the ground and the excited state radial wavefunctions. For both cases, we will give here a very simple analytic expression for the Franck-Condon factor that is in excellent agreement with fully quantal calculations. We will show that the Franck-Condon factor is proportional to |*Ψ*_g_(*R*_C_, *E*)|^2^, the square of the ground state wavefunction at the Condon point *R*_C_, where the quasimolecule is in exact resonance with the light. At *R*_C_ the difference between excited and ground potentials exactly matches *ℏω*. Our expression for the binary collision rate exhibits the proper quantum threshold law behavior as *E* → 0. It predicts that the binary collisional loss rate per atom, *γ*_binary_, also varies as $\Delta_{A}^{-2}$ and at typical BEC densities generally is comparable to or exceeds the atomic scattering rate, Eq. (1), for a wide range of blue detuning and greatly exceeds the atomic scatering rate when *ω* is tuned near a photoassociation resonance.

We will first describe the model and the basic properties of the ground state wavefunction. Then we will discuss the binary collision rates for the case of blue detuning and the reflection approximation for the free-free Franck-Condon factors. Then we will describe the modifications needed to describe the case of red detuning and free-bound Franck-Condon factors. Finally, we will consider some of the implications for a cold atomic gas near BEC.

## 2. Model

The binary collision rates for the two cases described in Fig. 1 will be expressed in terms of Franck-Condon factors between the ground and excited state wavefunctions. However, it is very desirable to formulate the problem using the field-dressed molecular picture of a collision in a light field. This procedure is well-documented elsewhere [22–27], even for cold collisions [28–30,5,14], and need not be described in detail here. A two-state model with a single ground state and a single molecular excited state will be assumed. In the weak-field limit it is straightforward to generalize to multichannel ground and excited states for real alkali systems with hyperfine structure. In fact, we have now developed computer codes that let us include both ground and excited state molecular hyperfine structure for alkali species for optical transitions in the weak field limit [12,13]. The ground state potential is *V*_g_(*R*) and the excited state potential is *V*_e_(*R*) − *ℏ* ***Δ***_A_, where both *V*_g_ and *V*_e_ → 0 as *R* → ∞. The ground and excited states are coupled optically by the interaction matrix element *ℏΩ*(*R*), where the molecular Rabi frequency is

$$\Omega \left(R\right)=\sqrt{2\pi I/c}\;d\left(R\right)=b\left(R\right)\Omega_{A},$$ (2)

where *d*(*R*) is the molecular transition dipole, and $0\leq b\left(R\right)\leq 2/\sqrt{3}$ relates the molecular and atomic Rabi frequencies. The particular value of *b* depends on the molecular states involved. The ground and excited dressed potentials cross at the Condon point *R*_C_, the point of quasimolecular resonance, where in the undressed picture of Fig. 1, the difference of upper and lower potential curves matches the photon energy *ℏω*:

$$\hbar \omega_{A}+V_{e}\left(R_{C}\right)-V_{g}\left(R_{C}\right)=\hbar \omega$$ (3)

$$V_{e}\left(R_{C}\right)-V_{g}\left(R_{C}\right)=\hbar \Delta_{A}.$$ (4)

In this paper we will carry out purely model illustrative calculations. For this purpose it is sufficient to use analytic Lennard-Jones potentials for the ground and excited states,

$$V_{i}\left(R\right)=4\varepsilon_{i}\left({\left(\frac{\sigma }{R}\right)}^{2n}-{\left(\frac{\sigma }{R}\right)}^{n}\right),$$ (5)

**Fig. 2:**
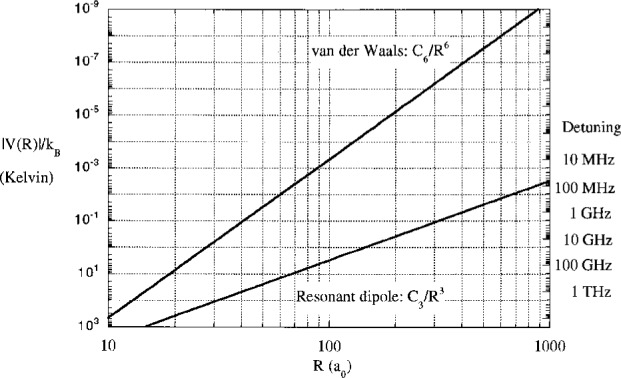
Magnitude of long range ground state van der Waals and excited state resonant dipole interaction potentials, shown using log scales. The interaction strengths were taken to be *C*_6_ = 1500 atomic units and *C*_3_ = 10 atomic units respectively, characteristic of Na atom interactions. The scale on the right hand side of the figure gives the detuning ***Δ***_A_ corresponding to the Condon points associated with the resonant dipole curve.

**Fig. 3:**
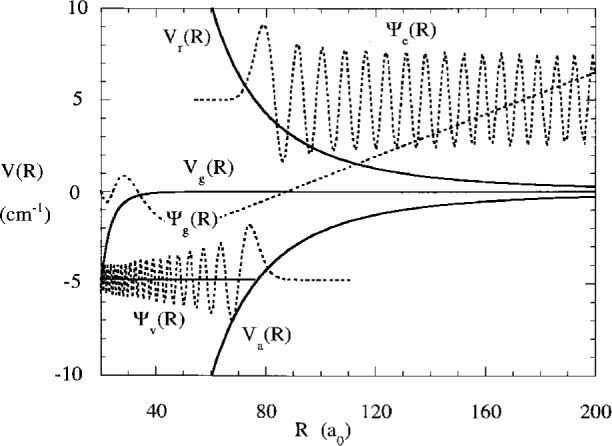
Long range ground and attractive and repulsive potentials on a linear scale for the same long range parameters as for Fig. 2. Typical free and bound excited state wavefunctions for a Na reduced mass are also shown, for an exit channel with 5 cm^−1^ kinetic energy and for a bound state with a binding energy of 4.87 cm^−1^. The 2 μK ground state wavefunction is nearly linear between 60 *a*_0_ and 200 *a*_0_.

*i* = *g* or *e* and *n* = 6 for the ground state van der Waals potential and *n* = 3 for the excited state resonant dipole potential. The long range variation is *C*_6_/*R*^6^ and *C*_3_/*R*^3^ respectively. We use a reduced mass characteristic of sodium. It is difficult to show on one graph the potentials over the very large range of energy and distance involved in cold collisions. Figure 2 shows on a logarithmic scale the magnitude |*V*(*R*)|/*k*_B_ (where *k*_B_ is the Boltzmann constant) of the long range potentials for the ground and excited states of the Na_2_ molecule, for which we take *C*_6_ = 1500 atomic units ( $e^{2}a_{0}^{5}$, where *e* = electron charge and *a*_0_ = Bohr radius = 0. 0529 nm) and |*C*_3_| = 10 atomic units ( $e^{2}a_{0}^{2}$). Figure 3 shows on a linear scale the ground and attractive and repulsive excited state potentials, as well as illustrative wavefunctions for each. Since the excited potential is orders of magnitude stronger than the ground state at long range, it is an excellent approximation to neglect the ground state contribution in calculating the Condon point:

$$\left|\Delta_{A}\right|=\frac{\left|C_{3}\right|}{{R_{C}}^{3}}\Rightarrow R_{C}={\left|\frac{C_{3}}{\Delta_{A}}\right|}^{1/3}.$$ (6)

Figure 2 also indicates the detunings associated with each *R*, taken as a Condon point. Note that the ground state interaction strength is not negligible at intermediate *R*, but is stronger than 1 μK whenever *R* is less than about 300 *a*_0_.

## 3. Ground State Wavefunction

### 3.1 Long Range Form

We need the ground state wavefunction to use in the reflection formula for the Franck-Condon factors we derive below. When the collision energy *E* is low enough, only collisions with zero relative angular momentum of nuclear motion contribute to scattering cross sections. The *s*-wave ground state wavefunction takes on the following well-known behavior as *R* → ∞:

$$\begin{array}{l} |\Psi_{g}^{+}\left(E\right)\rangle ∼e^{i\eta_{g}}{\left(\frac{2\mu }{\pi \hbar^{2}}\right)}^{1/2}\\ \frac{\sin\left(k_{\infty }R+\eta_{g}\right)}{\sqrt{k_{\infty }}}, \end{array}$$ (7)

where the $\sqrt{2\mu /\pi \hbar^{2}k_{\infty }}$ factor ensures energy normalization, ${\left|\langle \Psi_{g}^{+}\left(E\right)|\Psi_{g}^{+}\left(E^{\prime }\right)\rangle \right|}^{2}=\delta \left(E-E^{\prime }\right)$. The asymptotic kinetic energy is $E=\hbar^{2}k_{\infty }^{2}/2\mu$, where *μ* = *m*_A_/2 is the reduced mass, the deBroglie wavelength is *λ*_∞_ = 2*π*/*k*_∞_, and the velocity *υ*_∞_ = *ℏ k*/*μ*. It is sometimes more convenient to write the energy normalization factor as

$${\left(\frac{2\mu }{\pi \hbar^{2}k_{\infty }}\right)}^{1/2}=\frac{2}{{\left(h\upsilon_{\infty }\right)}^{1/2}}.$$ (8)

If *E* is small enough, the asymptotic phase *η*_g_ = *nπ − k*_g_*A*_s_, where *A*_s_ is the scattering length and *n* is the number of bound states supported by the ground state potential; the *nπ* term can be ignored, since it only introduces an inessential phase factor. The scattering length is a property of the whole potential and for real atoms is very sensitive to small uncertainties in the short range, chemical bonding, range of *R*. However, the scattering length provides a good parameter to characterize the effect on the long range wavefunction of the complete potential.

**Fig. 4:**
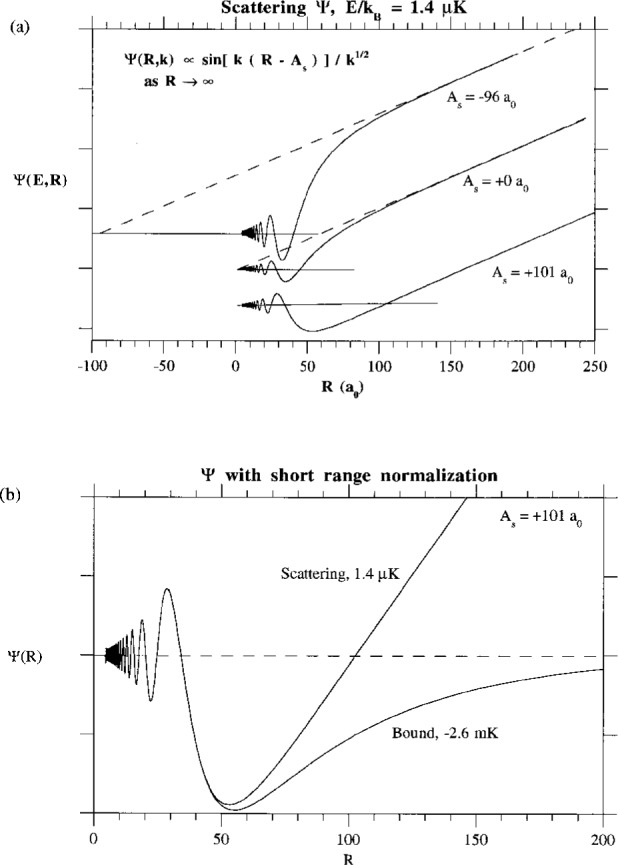
(a) Comparison of characteristic ground state near-threshold wavefunctions at 1.4 μK collision energy. The wavefunctions were calculated using a Na reduced mass and three different hypothetical model potentials which yield scattering lengths *A_s_* = − 96 *a*_0_, 0, *a*_0_, and + 101 *a*_0_. The intermediate range wavefunction projects to a node near *R* = *A*_s_. Of course, the scattering length for any real atomic species is fixed by the actual interaction potential and can not be varied (except possibly by introducing external fields). (b) Comparison of the scattering wavefunction for the potential for which *A_s_* = + 101 *a*_0_ with the last bound state wavefunction (bound state binding energy is 2.6 mK), when both are given a common WKB normalization near the distance of the deepest part of the ground state potential.

Figure 4a illustrates typical ground state wavefunctions (in their real form without the trivial complex phase factor $e^{i\eta_{g}}$) with positive, zero, and negative scattering lengths. These were calculated by making slight changes in the model potential parameters. The inner region wavefunction, say for *R* < 30 *a*_0_, is characterized by rapid oscillations as the local kinetic energy increases due to the acceleration by the attractive ground state potential. The intermediate range wavefunction, say for 80 *a*_0_ < *R* < *λ*_∞_/4, is nearly linear in *R* and extrapolates to an intercept near *R* = *A*_s_:

$$\Psi_{g}^{+}\left(R,E\right)\approx e^{i\eta_{g}}{\left(\frac{2\mu }{\pi \hbar^{2}}\right)}^{1/2}\frac{k_{\infty }\left(R-A_{s}\right)}{\sqrt{k_{\infty }}}.$$ (9)

A much more accurate representation of the ground state wavefunction in the intermediate range is found by correcting for the variation in phase and amplitude due to the nonzero ground state potential. The details of this derivation, based on an approximate treatment of the rigorous Milne equation for the phase-amplitude form of the wavefunction, are given in Appendix A. The result is that a more correct form of the long range wavefunction is found from Eqs. (60), (69), and (72)

$$\begin{array}{l} \Psi_{g}^{+}\left(R,E\right)=e^{i\eta_{g}}{\left(\frac{2\mu }{\pi \hbar^{2}k_{\infty }}\right)}^{1/2}\\ \;\times a\left(R\right)\sin\left(k_{\infty }\rho \left(R\right)\right), \end{array}$$ (10)

where

$$a\left(R\right)=1-{\left(\frac{R_{B}}{R}\right)}^{4}$$ (11)

$$\rho \left(R\right)=R-A_{s}-\frac{2}{3}{\left(\frac{R_{B}}{R}\right)}^{4}R.$$ (12)

**Fig. 5:**
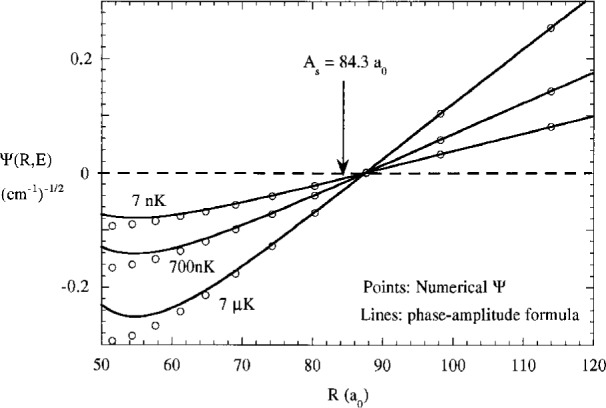
(a) Comparison of the exact calculated ground state wavefunction (points) with the predictions of the phase amplitude approximation, Eq. (10), using a Na reduced mass and a potential for which *A_S_* = 84.3 *a*_0_. The actual node position at *R* = 87.5 *a*_0_ is shifted to larger *R*. The node position is independent of collision energy, as predicted by Eq. (12).

Equation (10) only applies in the region *R* ≫ *R*_B_ so that the correction term (*R*_B_/*R*)^4^ ≪ 1, where

$$R_{B}={\left(\frac{\mu C_{6}}{10\hbar^{2}}\right)}^{1/4}$$ (13)

is a constant independent of energy with dimensionality of distance. It has a value of 42 *a*_0_ for Na and 77 *a*_0_ for Rb. Figure 5 compares the exact wavefunction and the result of Eq. (10) in the near linear region between 60 *a*_0_ and 120 *a*_0_ for a model Na potential with *A*_s_ = + 84.3 *a*_0_. Such a magnitude of *A*_s_ is near the actual value for doubly spin-polarized Na [31,32,12]. The actual wavefunction node at 87.5 *a*_0_ is shifted 3.2 *a*_0_ further out than *A*_s_, as accurately predicted by Eqs. (10) and (74). A much larger shift of 18 *a*_0_ in node position is predicted for ^87^Rb.

### 3.2 Short Range Form

Before leaving this discussion of the ground state wavefunction, it is useful to comment on the nature of the short range wavefunction as *E* → 0. When the collision energy *E* decreases towards zero, there will always be a characteristic quantum threshold connection between the asymptotic and short range wavefunction. It is possible to define a characteristic distance *R_Q_* where the WKB connection between the asymptotic and short range wavefunctions strongly breaks down for collision energies *E* < *E_Q_* [33, 34]. A necessary condition for onset of the threshold law behavior is *E* ≪ *E_Q_*. The values of *R_Q_* and *E_Q_* are determined by requiring that the maximum in the function d*λ* (*R*, *E*)/d*R* satisfy the following condition:

$$\frac{d\lambda \left(R_{Q},E_{Q}\right)}{dR}=\frac{1}{2}.$$ (14)

where the local deBroglie wavelength *λ* (*R*, *E*) is defined by Eq. (63) in the Appendix A. For all *E* < *E_Q_* there is a range of distances around *R_Q_* where d*λ* (*R*, *E*)/d*R* > 1/2 and the WKB approximation fails. Using Eqs. (17) and (18) of [34],

$$R_{Q}=\frac{20^{1/4}}{2^{1/2}{\left(7a\right)}^{1/6}}R_{B}=1.043R_{B},$$ (15)

where $a=\frac{1}{27}{\left(\frac{14}{3}\right)}^{3}{\left(\frac{7}{2}\right)}^{1/2}=7.0419$. Thus, we find that *R*_B_ = 0.96*R_Q_*, independent of potential parameters (since both have identical scalings with *C*_6_ and *μ*), and the defined parameters *R*_B_ and *R_Q_* turn out to be fortuitously very close in magnitude. Since each of these only defines a qualitative range of distance associated with a change in the wavefunction, they can be used interchangably. The WKB breakdown region is actually very broad (An example for a He mass is in Fig. 3 of Ref. [33]), and becomes broader as energy is lowered below *E_Q_* = 19 mK for Na or *E_Q_* = 1.5 mK for Rb [34]. Therefore, significant departure from the WKB form begins at distances well to the right of *R_Q_*, and Eqs. (10) and (11) show that the amplitude of the intermediate range wavefunction remains much closer to its asymptotic amplitude $\propto 1/\sqrt{k_{\infty }}$ than to the local WKB amplitude $\propto 1/\sqrt{k\left(R,E\right)}$. Thus, the amplitude of the ground state wavefunction does not appreciably exhibit the acceleration due to the ground state potential unless *R* is on the order of or less than *R*_Q_ ≈ *R*_B_. This is one of the reasons why a semiclassical analysis fails in the long range region as *E* → 0.

The form of the wavefunction in Eq. (60) is completely rigorous at all distances and energies, providing that the Milne equation described in Appendix A is solved exactly. References [33, 34] show that the ground state wavefunction for the inner region *R* ≪ *R_Q_* is well approximated by the form

$$\begin{array}{l} \Psi_{g}^{+}\left(R,E\right)={\left(A_{i}k_{\infty }\right)}^{1/2}e^{i\eta_{g}}{\left(\frac{2\mu }{\pi \hbar^{2}}\right)}^{1/2}a_{g}^{\text{WKB}}\left(R,E\right)\\ \;\times \sin\left(\beta_{g}^{\text{WKB}}\left(R,E\right)\right) \end{array}$$ (16)

$$={\left(A_{i}k_{\infty }\right)}^{1/2}\Psi_{g}^{\text{WKB}+}\left(R,E=0\right),$$ (17)

where *A_i_* is an energy-independent constant having the dimension of length and $a_{g}^{\text{WKB}}\left(R,E\right)=1/\sqrt{k_{g}\left(R,E\right)}$ is the WKB amplitude. Equation (16) shows that the overall *shape* of the inner range wavefunction, $\Psi_{g}^{\text{WKB}+}\left(R\ll R_{Q}\right)$, is independent of energy, whereas the overall amplitude decreases as (*A_i_k*_∞_)^1/2^ as *E* → 0. This is illustrated in Fig. 4b, which shows a scattering wavefunction near threshold and the wavefuncton for the last bound state, both normalized at short range to the same WKB form. Scattering wavefunctions near threshold, as well as the last bound state wavefunction, when given this common normalization, are almost indistinguishable for *R* < *R_Q_*. The physical reason for this is that the local amplitude and phase are determined by the ground state potential in a region where it is very deep compared to the initial collision energy or bound state binding energy.

Both the inner range and outer range approximate wavefunctions, Eqs. (16) and (10), are proportional to $\sqrt{k_{\infty }}$, in accordance with the threshold law requirement that the ground state wavefunction for *R* ≪ 1/*k*_∞_ everywhere vanishes as $\sqrt{k_{\infty }}$. This ensures that the probability for inelastic processes due to the ground state entrance channel vanish as *k*_∞_ as *E* → 0. This is the basic threshold law for inelastic collision rates which must be satisfied for light-induced inelastic events for either red or blue detuning.

## 4. Collisions for Blue Detuning

The case of cold collisions in a blue detuned light field has been extensively studied recently, both experimentally and theoretically [14,15,9,35–41]. Figure 4a shows the ground and excited state potentials for the case of a very large detuning of 5 cm^−1^, or 300 GHz, from atomic resonance. If the light intensity is strong enough (this will be defined more precisely below), the ground state atoms approaching one another encounter an effective repulsive interaction near the Condon point *R*_C_ and are repelled. This gives rise to the phenomenon of optical shielding [15,9,35–41], by which the light prevents the atoms from approaching closer than *R*_C_, thereby reducing the rate of ground state processes which require the atoms to be closer together than *R*_C_. The light also modifies the ground state elastic scattering rate and scattering length, and results in the creation of hot excited state atoms which share the kinetic energy released, *ℏ****Δ***_A_ [14,15,9]. Since we are considering the weak field limit here, the shielding effect is small and we will concentrate on the latter energy release process. The event rate coefficient for this process is

$$K_{e}\left(\Delta_{A}\right)=\langle \frac{\pi \upsilon_{g}}{k_{g}^{2}}P_{e}\left(E,\Delta_{A}\right)\rangle =\langle \frac{\pi \hbar }{\mu k_{g}}P_{e}\left(E,\Delta_{A}\right)\rangle ,$$ (18)

where the brackets imply an average over the distribution of ground state velocities *υ*_g_, and

$$P_{e}\left(E,\Delta_{A}\right)={\left|S_{\text{eg}}\left(E,\Delta_{A}\right)\right|}^{2}$$ (19)

is the probability for the event by which two ground state atoms collide in a light field and produce one excited and one ground state atom, both of which have enough kinetic energy to escape any weak trapping potential.

When the radiative coupling is small, the radiative distorted wave approximation [24,26,27], can be used for the *S*-matrix element:

$$S_{\text{eg}}\left(E,\Delta_{A}\right)=-2\pi i\langle \Psi_{e}^{-}\left(E+\hbar \Delta_{A}\right)\left|V_{\text{eg}}\left(R\right)\right|\Psi_{g}^{+}\left(E\right)\rangle ,$$ (20)

where *V*_eg_(*R*) = *ℏΩ*(*R*) from Eq. (2). The kinetic energy in the asymptotic ground and excited state channels are *E* and *E* + *ℏ****Δ***_A_ respectively. The asymptotic excited state wavefunction is:

$$\left|\Psi_{e}^{-}\left(E,\Delta_{A}\right)\right|\rangle =∼e^{i\eta_{e}}{\left(\frac{2\mu }{\pi \hbar^{2}k_{e,\infty }}\right)}^{1/2}\sin\left(k_{e,\infty }R+\eta_{e}\right).$$ (21)

**Fig. 6:**
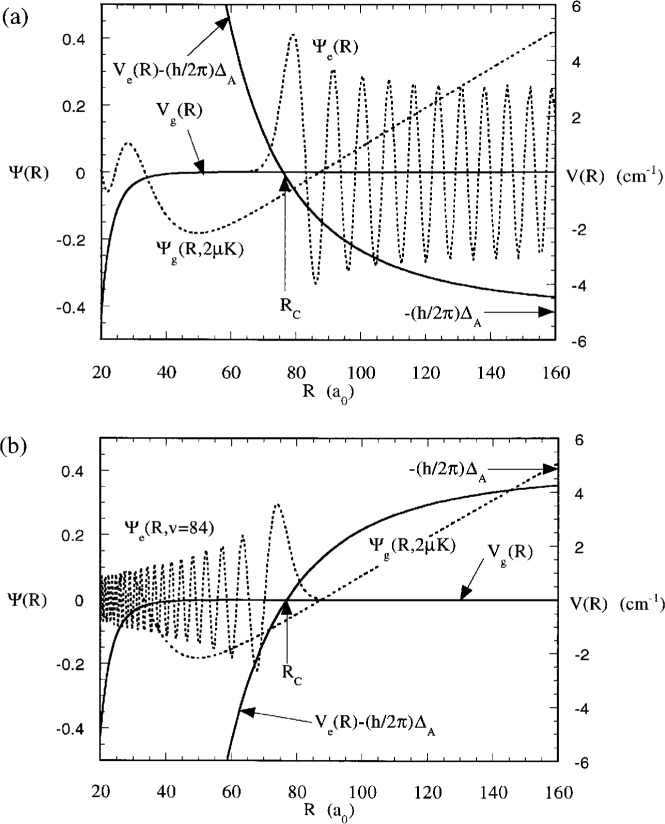
(a) Ground and repulsive excited state field-dressed potentials for a blue detuning ***Δ***_A_ of 5 cm^−1^ = 300 GHz and laser intensity *I* = 0. The crossing at *R* = *R*_C_ becomes an avoided crossing when *I ≠* 0. Ground and excited state wavefunctions are also shown. (b) Similar figure for the case of a red detuning ***Δ***_A_ of −4.87 cm^−1^, in resonance with the *υ* = 84 level of the attractive excited potential.

The ground and excited state wavefunctions are also illustrated in Fig. 6a. The excited state wavefunction oscillates much more rapidly than the ground state one because of the much greater kinetic energy in the exit channel.

By assuming that the radiative coupling *V*_eg_(*R*) is either independent of *R*or slowly varying with *R* near *R*_C_, then this term can be removed from the integrand in Eq. (20), giving the radiative distorted wave result that

$$P_{e}\left(E,\Delta_{A}\right)=4\pi^{2}V_{C}^{2}\left|\langle \Psi_{e}^{-}\left(E,\Delta_{A}\right)\right|{\Psi_{g}^{+}\left(E\right)\rangle |}^{2}$$ (22)

$$=4\pi^{2}V_{C}^{2}F_{\text{eg}}\left(E,\Delta_{A}\right),$$ (23)

where *V*_C_ = *V*_eg_(*R*_C_) and

$$F_{\text{eg}}\left(E,\Delta_{A}\right)=\left|\langle \Psi_{e}^{-}\left(E,\Delta_{A}\right)\right|{\langle \Psi_{g}^{+}\left(E\right)\rangle |}^{2}$$ (24)

is a free-free Franck-Condon factor. Although direct evaluation of *F*_eg_ by numerical quadrature does not converge, since both wavefunctions oscillate with finite amplitude to *R* = ∞, there are several methods available for evaluating *F*_eg_ numerically [27], as well as approximate formulas based on the local nature of the integrand near the Condon point *R*_C_. Here we use a two-state close coupled scattering calculation of the collision in a weak radiation field with an *R*-independent Rabi frequency, and extract *S*_eg_(*e*) from the asymptotic field-dressed wavefunction [24–27]. Then the Franck-Condon factor, independent of the laser intensity chosen for the calculation, is calculated from:

$$F_{\text{eg}}\left(E,\Delta_{A}\right)=\frac{{\left|S_{\text{eg}}\left(E,\Delta_{A}\right)\right|}^{2}}{4\pi^{2}V_{C}^{2}},$$ (25)

where *S*_eg_(*E*, ***Δ***_A_) is calculated using a standard close coupling scattering code. This method can be generalized to a multichannel version when there are more than two channels [27], and thus could be used for atoms with hyperfine structure [12, 13].

**Fig. 7:**
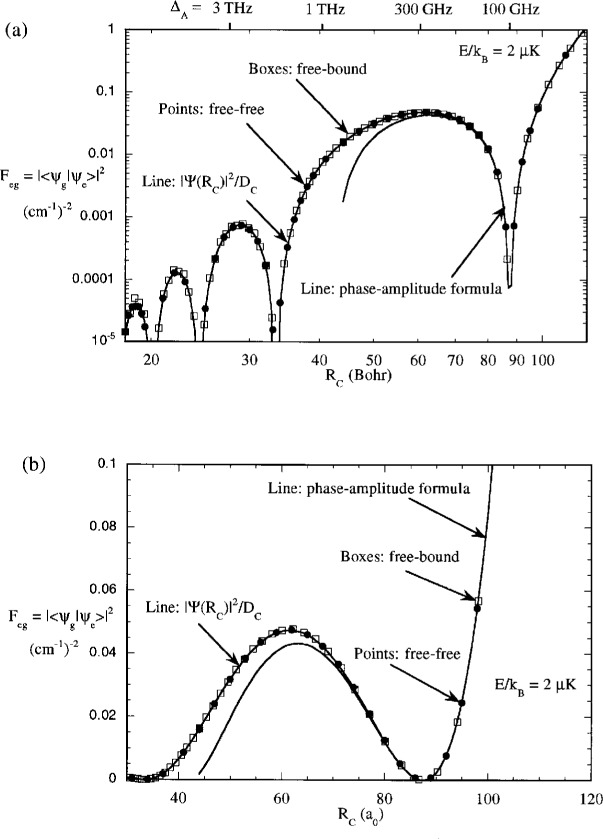
Comparison of exact and approximate evaluations of the Franck-Condon factors for different Condon points *R*_C_. The correspondance of detuning to *R*_C_ is indicated on the top horizontal axis. The free-free Franck-Condon factors have dimensionality of (energy)^22^, where here energy is expressed in cm^−1^ units. Since the same magnitude of the *C*_3_ coefficient was use for the repulsive and attractive curves in our model calculations, the exact free-bound Franck-Condon factors are seen to lie on nearly the same curve as the free-free ones, when the former are divided by ∂*E_υ_* /∂*υ* to normalize them per unit energy. This is as predicted by Eqs. (31) and (41). The reflection approximation formula of Eq. (31), |*Ψ*_g_ (*R*_C_)|^2^/*D*_C_, is in excellent agreement with the exact calculations over the whole range shown. The intermediate range phase-amplitude formula, Eq. (32), is good agreement for *R* > 70 *a*_0_. (a) logarithmic scale (b) linear scale showing region near outer nodes.

Figure 7 shows *F*_eg_ calculated for collision energy *E*/*k*_B_ = 2 *μ*K using a model ground state potential with the reduced mass for Na collisions and a calculated scattering length of +84.3 *a*_0_. The detuning range corresponding to *R* = 20 *a*_0_ to 200 *a*_0_ is 8 THz to 8 GHz. The Franck-Condon pattern shows an oscillatory pattern versus *R*_C_ or detuning.

## 5. Reflection Approximation

We will now turn our attention to developing approximations that will give considerable physical insight into the nature of light scattering at very low collision energy. Let us first look at semiclassical approximations. We will assume a single Condon point *R*_C_. Thus, our analysis does not apply to states with significant contributions from two Condon points, such as the low vibrational levels of the $0_{g}^{-}$ state of Na_2_ [12, 13]. A semiclassical approximation for the free-free Franck-Condon factor $F_{\text{eg}}^{\text{SC}}\left(E\right)$ is given by Eq. (46) of the reference [42] (we remove the uninteresting $V_{C}^{2}$ factor):

$$F_{\text{eg}}^{\text{SC}}\left(E,\Delta_{A}\right)=\frac{2}{h\upsilon_{C}d_{C}},$$ (26)

where

$$D_{C}={\left|\frac{d}{dR}\left(V_{e}\left(R\right)-V_{g}\left(R\right)\right)\right|}_{R=R_{C}}$$ (27)

$$\approx \frac{3C_{3}}{{R_{C}}^{4}}=\frac{3}{R_{C}}\left|\Delta_{A}\right|$$ (28)

is the difference in potential slopes evaluated at *R*_C_ and $\upsilon_{C}=\upsilon_{g}\left(R_{C}\right)=\sqrt{2(E-V_{g}\left(R_{C}\right)/\mu }$ is the local velocity at *R*_C_. The second equation follows from neglecting *V*_g_ in comparison to *V*_e_. The corresponding semiclassical treatment in the field-dressed picture of Fig. 6a is given by the Landau-Zener (LZ) approximation [14,15,9] for *P*_e_:

$$P_{e}^{\text{LZ}}\left(E,\Delta_{A}\right)=2\;e^{-A_{C}}\left(1-e^{-A_{C}}\right)\approx 2\;A_{C}.$$ (29)

The latter approximation applies when the dimensionless LZ adiabaticity parameter

$$A_{C}=\frac{4\pi^{2}V_{C}^{2}}{h\upsilon_{C}D_{C}}$$ (30)

is small compared to unity. This condition also ensures the low intensity regime of linear variation of *P*_e_ with light intenstiy. Using Eq. (25) to obtain $F_{\text{eg}}^{\text{SC}}\left(E\right)$ from Eq. (29) gives exactly the same expression as in Eq. (26). Note that both Eqs. (26) and (30) have left out the phase factor sin^2^(*β*_g_(*R*_C_) − *β*_e_(*R*_C_)). Actually, the phase factor has been replaced by its average value, 1/2. Note that $P_{e}^{\text{LZ}}$ given by Eq. (29), even with the phase factor, does not satisfy the threshold law requirement to vary as *k*_∞_ as *E* → 0. In fact, $F_{\text{eg}}^{\text{SC}}\left(E\right)$ from Eq. (26) is in very poor agreement with the quantum mechanical *f*_eg_(*E*) in Fig. 7 and is not shown in the figure.

The usual stationary phase derivation of Eq. (26) can be adapted to give the correct threshold law as *E* → 0. The details are given in Appendix B. The result is a key result of this paper, a simple reflection formula:

$$F_{\text{eg}}^{R}\left(E,\Delta_{A}\right)=\frac{1}{D_{C}}{\left|\Psi_{g}^{+}\left(R_{C},E\right)\right|}^{2}.$$ (31)

The Franck-Condon factor is directly proportional to the square of the ground state wavefunction. It only depends on the excited state through the slope term *D*_C_. This expression explicitly satisfies the threshold law, since at all distances *R* << λ_∞_, |*Ψ*_g_|^2^ is proportional to *k*_∞_ when *E* is sufficiently smaller than *E_Q_* [33, 34]. When *R* > *R_Q_*, the quantum wavefunction is well-approximated by Eqs. (10) – (12), and the intermediate range phase-amplitude approximation to $F_{\text{eg}}^{R}$ is:

$$F_{\text{eg}}^{PA}\left(E,\Delta_{A}\right)=\frac{4}{h\upsilon_{\infty }D_{C}}a_{C}^{2}\sin^{2}\left(k_{\infty }\rho_{C}\right).$$ (32)

where *a*_C_ = *a*(*R*_C_) and *ρ*C = *ρ*(*R*_C_). The basic difference between this formula and the Landau-Zener one, Eq. (26), other than the phase factor, is the presence of the asymptotic ground state velocity in the denominator instead of the local velocity *υ*_C_. If *R*_C_ is in the range *R_Q_* << *R*_C_ << λ_∞_/2 so that the sin function is linear in its argument, then

$$F_{\text{eg}}^{PA}\left(E,\Delta_{A}\right)=\frac{2\mu a_{C}^{2}\rho_{C}^{2}}{\pi \hbar^{2}D_{C}}k_{\infty }.$$ (33)

The equivalent approximation for *P*_e_ is found from Eq. (23),

$$P_{e}\left(E,\Delta_{A}\right)=\frac{16\pi^{2}V_{C}^{2}}{h\upsilon_{\infty }D_{C}}a_{C}^{2}k_{\infty }^{2}\rho_{C}^{2}.$$ (34)

**Fig. 8:**
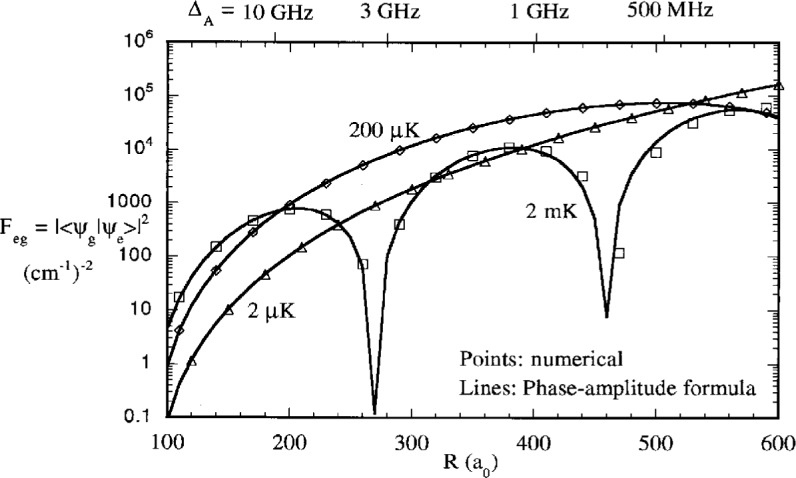
Comparison of the exact free-free Franck Condon factors and the predictions of the phase-amplitude formula, Eq. (32), for higher collision energies, *E*/*k*_B_ = 2 μK, 200 μK, and 2000 μK = 2 μK. The breakdown of the reflection approximation is seen at the larger *R*_C_ for 2000 μK.

Figure 7 also shows the predictions of the *F^R^* and *F^PA^* formulas compared to the exact results for 2 μK collisions. The full reflection formula is an excellent approximation over a wide range of Condon points, and even remains very good into about 20 *a*_0_ where the ground state has accelerated the atoms to a velocity much higher than their initial one. The *F^PA^* approximation formula is also excellent at intermediate range, until *R* begins to approach *R*_B_ ≈ *R_Q_*. Figure 8 compares the exact and *F*^PA^ results for other collision energies up to 2 mK. We see that the reflection approximation is very good for almost the whole range of temperatures encountered for trapped laser cooled atoms, including the *E* → 0 limit.

## 6. Collisions for Red Detuning

We can carry out a very similar treatment for the case of the free-bound Franck-Condon factor for red detuning. Figure 6b illustrates the dressed potentials and wavefunctions for a red detuning case. The basic difference from blue detuning is that the attractive potential supports discrete bound states. When the laser frequency *ω* is tuned near the position of a particular bound level with vibrational quantum number *υ*, the level can be excited and decay to some product *p*, which is no longer trapped. The decay process is primarily spontaneous emission leading to formation of molecular species or hot atoms [28, 42]. In the field dressed picture of Fig. 6b, the bound state is a scattering resonance embedded in the continuum very close to threshold. The rate coefficient for red detuning is also given by Eq. (18), but the *S*-matrix element is given by a modified resonant scattering form described by Napolitano et al. [5]:

$$\begin{array}{c} {\left|S_{\text{pg}}\left(E,\upsilon ,\Delta_{A}\right)\right|}^{2}\\ =\frac{\gamma_{p}\gamma_{s}\left(E,\upsilon ,\Delta_{A}\right)}{{\left(\frac{E-\Delta_{v}}{\hbar }\right)}^{2}+{\left(\gamma_{v}/2\right)}^{2})}, \end{array}$$ (35)

where ***Δ***_v_ is the detuning relative to the position *E*_v_ of the bound state. The total decay rate of the excited bound state *υ* is *γ_υ_* = *γ*_p_ + *γ_s_* (*E*, *υ*, ***Δ***_A_) + *γ*_o_, where *γ*_p_ is the rate by which the bound state resonance decays to the detected product, *γ_s_* (*E*, *υ*, ***Δ***_A_) is the stimulated emission rate back to the ground state continuum, and *g*o is the decay rate due to any other undetected processes (such as molecular predissociation). The critical element for our purposes is the *γ_s_* (*E*, *υ*, ***Δ***_A_) factor, which describes how the cold colliding atoms couple to the excited bound state. For low light intensity, this factor is given by the Fermi golden rule expression [5]:

$$\gamma_{s}\left(E,\upsilon ,\Delta_{A}\right)=\frac{2\pi }{\hbar }\left|\langle \Psi_{e}\left(\upsilon \right)\right|V_{\text{eg}}\left(R\right){\left|\Psi_{g}^{+}\left(E\right)\rangle \right|}^{2},$$ (36)

where *Ψ*_e_(*υ*) is the unit-normalized bound state wave-function of the excited bound state. Using the same factorization approximation as for Eq. (23), this can be written in terms of a bound-free Franck-Condon factor,

$$\begin{array}{c} \gamma_{s}\left(E,\upsilon ,\Delta_{A}\right)=\\ \frac{2\pi }{\hbar }V_{C}^{2}\left|\langle \Psi_{e}\left(\upsilon \right)\right|{\Psi_{g}^{+}\left(E\right)\rangle |}^{2} \end{array}$$ (37)

$$=\frac{2\pi }{\hbar }V_{C}^{2}F_{\text{eg}}\left(E,\upsilon ,\Delta_{A}\right).$$ (38)

The assumption is that $V_{C\;}^{2}$ is evaluated at a single Condon point; if there is more than one Condon point, the interference between the different amplitudes from each Condon point would need to be taken into account.

The derivation of the reflection approximation formula for the bound-free Franck-Condon factor *F*_eg_(*E*, υ, ***Δ***_A_) follows immediately from the same treatment in Appendix B that we used in deriving the free-free Franck-Condon factor. The essential difference is that the unit-normalized bound state wavefunction can also be written in energy-normalized phase-amplitude form just like Eq. (16), as is commonly done in generalized multichannel quantum defect theory [44]. It is only necessary to introduce the vibrational spacing

$$\frac{\partial E_{\upsilon }}{\partial \upsilon }=h\nu_{\upsilon },$$ (39)

where *ν_υ_* is the vibrational frequency, the number of complete vibrational cycles per unit time. Then the unit normalized bound state wavefunction has the form [44]

Ψe(R,υ)=(∂Eυ∂υ)1/2(2μπℏ2)1/2αe(R,υ)sin(βe(R,υ)). (40)

where *α* and β can be determined quantum mechanically from Eqs. (61) and (62), or alternatively, from the WKB form, Eq. (64). The derivation in Appendix A, adapted for a right hand turning point, carries through to give

$$F_{\text{eg}}\left(E,\upsilon ,\Delta_{A}\right)=\frac{\partial E_{\upsilon }}{\partial \upsilon }\frac{1}{D_{C}}{\left|\Psi_{g}^{+}\left(R_{C},E\right)\right|}^{2},$$ (41)

where $\Psi_{g}^{+}\left(R_{C},E\right)$ can be calculated exactly or taken from Eq. (10)) above. The vibrational spacing function can be evaluated approximately from the discrete level spacing,

$$\frac{\partial E_{\upsilon }}{\partial \upsilon }\approx \frac{E_{\upsilon +1}-E_{\upsilon -1}}{2},$$ (42)

or more rigorously from the wavefunction at some short range point *R*_0_:

$$\frac{\partial E_{\upsilon }}{\partial \upsilon }=\frac{\pi \hbar^{2}}{2\mu }\left(k_{0}{\left|\Psi_{e}\left(R_{0},\upsilon \right)\right|}^{2}+\frac{1}{k_{0}}{\left|\frac{d\Psi_{e}\left(R_{0},\upsilon \right)}{dR}\right|}^{2}\right),$$ (43)

where *k*_0_ = *k*_e_(*R*_0_). This equation immediately follows from Eq. (40) upon using the WKB forms in Eqs. (77)–(78). The result depends only weakly on the choice of *R*_0_ as long as it is in the classical region of the potential well. We use Eq. (43) in the numerical results described below.

Although the free-free and free-bound Franck-Condon factors have different dimensionality, they may be compared by evaluating the free-bound Franck-Condon factor per unit energy, or *F*_eg_(*υ*, ***Δ***_A_)(∂*E_υ_* /∂*υ*)^−1^. Figure 7 also shows the results of evaluating this function for a number of discrete bound levels using an attractive potential that has the same magnitude of the *C*_3_ long range potential coefficient as for the repulsive state (see Fig. 3). Each bound level defines a distinct Condon point nearly equal to the outer classical turning point. The free-bound and free-free Franck-Condon factors fall on almost exactly the same curve in Fig. 7, even when *R*_C_ is as small as 20 *a*_0_. This is expected from the fact that the free-free and free-bound transitions in the model calculation have the same ground state wave-function and *D*_C_ at the same Condon points. The results in Fig. 7 show that the reflection approximation is just as good for bound states as for free states.

Now we can easily derive from Eqs. (38) and (41) a simple expression for the stimulated decay rate of the excited bound state to the ground continuum, *γ_s_* (*E*, ***Δ***_A_):

$$\gamma_{s}\left(E,\Delta_{A}\right)=\frac{2\pi }{\hbar }\frac{V_{C}^{2}}{D_{C}}h\nu_{\upsilon }{\left|\Psi_{g}^{+}\left(R_{C},E\right)\right|}^{2}$$ (44)

$$=\frac{2\pi }{\hbar }\frac{V_{C}^{2}}{D_{C}}h\nu_{\upsilon }\frac{4}{h\upsilon_{\infty }}a_{C}^{2}\sin^{2}\left(k_{\infty }\rho_{C}\right)$$ (45)

$$=\left\{\frac{16\pi^{2}V_{C}^{2}}{h\upsilon_{\infty }D_{C}}a_{C}^{2}k_{\infty }^{2}\rho_{C}^{2}\right\}\upsilon_{b}$$ (46)

$$=P_{e}\left(E\right)\upsilon_{b}.$$ (47)

The probability *P*_e_(*E*) in Eq. (47), defined by the expression in braces in Eq. (46), is *identical* in form to the probability in Eq. (34) for a free-free transition. The expression in Eq. (47) thus has a pleasing physical interpretation: the total rate of decay out of the bound state is the probability of decay during one vibration cycle (corresponding to a “complete” collision with incoming and outgoing parts) times the frequency of vibration (number of cycles per second). The overall probability for the photoassociation transition, given by the resonance scattering expression in Eq. (35), explicitly exhibits the threshold law form due to the *γ_s_* factor in the numerator.

The form in Eq. (44), proportional to the square of the ground state wavefunction, will also give the threshold law form for *p*- and *d*-waves, etc. It also explicitly shows why the photoassociation spectra map out the nodal structure of the ground state wavefunction in the intensity pattern versus vibrational quantum number [28, 17]: as detuning ***Δ***_A_ changes from level to level, the Franck-Condon factor tracks the ground state wavefunction at the changing *R*_C_. This effect is expected to be much cleaner in very low temperature spectra, where only *s*-waves contribute, than in most of the existing higher temperature experiments, where *s*-, *p*-, *d*-waves, etc., all may contribute. The *p*- and *d*-waves do not generally have the same nodal structure in the intermediate range as the *s*-wave does.

## 7. Collision Rates Near BEC

In order to estimate the effect of binary collisions in a cold dense atomic gas, it is necessary to compare the rate for the binary process with the atomic scattering rate *γ*_atomic_ in Eq. (1). The binary event rate is

$$\gamma_{\text{binary}}=K_{e}\left(\Delta_{A}\right)N,$$ (48)

where *K*_e_ is given by Eq. (18). For the purpose of estimating the relative importance of atomic and binary light scattering events in causing loss of trapped atoms due to heating, it is necessary to compare *γ*_atomic_ and 2*γ*_binary_, since two atoms are lost per binary event. For the large detuning and moderate density conditions we consider, the influence of any collective effects on light scattering is not likely to alter significantly these basic magnitudes. We will base our estimates on the intermediate range formulas for *R* > *R*_B_, Eqs. (34) and (46), since these apply to a wide range of detuning from near resonance to beyond 100 GHz.

Using Eqs. (34) in (18), the rate coefficient for trap loss events for blue detuning in the intermediate range is

$$2K_{e}^{\text{blue}}\left(\Delta_{A}\right)=\frac{32\pi^{3}V_{C}^{2}}{hD_{C}}a_{C}^{2}\rho_{C}^{2}.$$ (49)

This expression is independent of collision temperature, as is should be. Using Eqs. (2), (6) and (28) and the fact that we can write [43, 42]

$$C_{3}=f_{3}\hbar \gamma_{A}{\left(\frac{\lambda_{A}}{2\pi }\right)}^{3},$$ (50)

the rate coefficient can be algebraically transformed to the form

$$2K_{e}^{\text{blue}}\left(\Delta_{A}\right)=\frac{2b_{C}^{2}f_{3}}{3\pi }\gamma_{A}{\left(\frac{\Omega_{A}}{\Delta_{A}}\right)}^{2}\frac{1}{N_{\lambda }}f_{C}.$$ (51)

In Eq. (50)*f*_3_ is a fraction of order unity depending on the particular molecular states involved (In Hund’s case (a) it is 1/2 for a Σ state and 1/4 for a P state; in general it will be a function of *R* that can be worked out for any particular state). The following definitions are used in Eq. (51):

$$b_{C}=b(R_{C})$$ (52)

$$N_{\lambda }=\frac{1}{{\lambda_{A}}^{3}}$$ (53)

$$f_{C}={\left(1-{\left(\frac{R_{B}}{R_{C}}\right)}^{4}\right)}^{2}{\left(1-\frac{A_{s}}{R_{C}}-\frac{2}{3}{\left(\frac{R_{B}}{R_{C}}\right)}^{4}\right)}^{2}.$$ (54)

Recall that *b*(*R*) is defined by Eq. (2). The $2b_{C}^{2}f_{3}/3\pi$ factor in Eq. (51) is a dimensionless molecular physics factor depending on the specific transitions involved, and it has a maximum value of

$$\frac{4}{9\pi }\approx \frac{1}{7}.$$

The density *N_λ_* corresponds to one atom per cubic *λ*_A_. The density near BEC conditions in a cold atomic gas is typically larger than *N_λ_*. The factor *f*_C_ has a nominal order of magnitude unity and reflects the phase of the ground state wavefunction. It will have a node near *A*_s_ if *A*_s_ is positive and in the intermediate range.

Using Eq. (51), we find

$$\frac{2\gamma_{\text{binary}}^{\text{blue}}}{\gamma_{\text{atomic}}}\approx \frac{1}{7}\frac{N}{N_{\lambda }}f_{C}.$$ (55)

This fraction is nearly independent of the blue detuning ***Δ***_A_ over a wide range, since *f*_C_ ≈ 1 (except near the node). Since *N_λ_* = 5×10^12^ cm^−3^ for Na and 2310^12^ cm^−3^ for Rb, we see that the order of magnitude of the light-induced binary collision loss rate is comparable to or larger than the atomic light scattering rate when the density is in the range of 10^13^ to 10^14^ cm^−3^.

It is also possible to get a simple expression for the rate coefficient for trap loss due to a bound state photo-association resonance by using Eqs. (47) and (35) in Eq. (18). The thermal average can be greatly simplified at very low *T* when *k*_B_*T* is very small compared to the linewidth *γ_υ_* of the bound state photoassociation resonance *υ*. Since the numerator of the resonance scattering *S*-matrix element, Eq. (35), satisfies the threshold law form, the energy-dependence disappears in the numerator of the resonance scatttering rate coefficient expression. Then we are left only with the energy dependence in the *E* − ***Δ****_υ_* term in the denominator of Eq. (35). But since *k*_B_*T* is small compared to *either* of the terms in the denominator, the *E* dependence is negligible and can be dropped. We also assume *γ_υ_* = *γ_p_*, that is, each decay results in a loss event, since the stimulated decay rate is assumed to be small (*γ*_s_ ≪ *γ_υ_*). Thus, the thermal average rate coefficient due to resonance *υ* becomes, in the *T* → 0 limit,

$$2K_{e}^{\text{red}}(\upsilon )=\left(\frac{\pi \upsilon_{\infty }}{k_{\infty }^{2}}\gamma_{s}(\upsilon ,\Delta_{A})\right)\frac{\gamma_{p}}{{\left(\Delta_{\upsilon }\right)}^{2}+{\left(\gamma_{\upsilon }/2\right)}^{2}}$$ (56)

$$=2K_{e}^{\left(1\right)}\frac{v_{\upsilon }\gamma_{p}}{{\left(\Delta_{\upsilon }\right)}^{2}+{\left(\gamma_{\upsilon }/2\right)}^{2}}.$$ (57)

In Eq. (57)

$$2K_{e}^{\left(1\right)}(\Delta_{A})=\frac{32\pi^{3}V_{C}^{2}}{hD_{C}}a_{C}^{2}\rho_{C}^{2}.$$ (58)

is identical in form to $2K_{e}^{\text{blue}}$ from Eq. (49). It is the *single-cycle* loss rate coefficient, due to passing the crossing once in each direction. The net rate is determined by the resonance expression. We can envision two possible limits. The first is the exact resonance case when the laser is tuned to the bound level so ***Δ***_v_ = 0. In this case, the resonance enhancement factor over the single pass result reduces to 4*ν_υ_τ_υ_*, where *τ_υ_* = 1/*γ_υ_* is the decay time of level *υ*. But *ν_υ_τ_υ_* is just the number *N_υ_* of complete cycles in one lifetime. So the maximum loss rate coefficient is $2K_{e}^{\max}(\upsilon )=8K_{e}^{\left(1\right)}N_{\upsilon }$. The second limit is when the detuning is as far as possible from resonance. Assuming equal spacing of vibrational levels, the largest detuning is just half of the vibrational spacing; thus, the maximum detuning is just ***Δ****_υ_* = *πτ_υ_* ≫ *γ_υ_*. This gives 1/(*π*^2^*N_υ_*) ≪ 1 for the factor multiplying $2K_{e}^{\left(1\right)}$ in Eq. (57). The minimum value the rate coefficient can take is $2K_{e}^{\min}=2K_{e}^{\left(1\right)}/(\pi^{2}N_{\upsilon })$. Of course, we must add the off-resonance contributions of all levels *υ* − 1, *υ* + 1, etc., to get the total of *f*-resonant rate coefficient. If the vibrational spacing is assumed to be uniform, the minimum rate coefficient from summimg over all such contributions is $\approx 2K_{e}^{\left(1\right)}/(4N_{\upsilon })$. Thus, we estimate

$$\frac{1}{4N_{\upsilon }}(2K_{e}^{\left(1\right)})<(2K_{e}^{\text{red}})<(4N_{\upsilon })(2K_{e}^{\left(1\right)}).$$ (59)

From Eq. (59) and (55), and the fact that *N_υ_* ≫ 1 whenever ***Δ***_A_ ≫ *γ*_A_, we see that $2\gamma_{\text{binary}}^{\text{red}}$ will be a sharply peaked function that is much smaller than $2\gamma_{\text{binary}}^{\text{blue}}$ (for the same |***Δ***_A_|) when *ω* is tuned between resonances, but which is much larger than $2\gamma_{\text{binary}}^{\text{blue}}$ when the *ω* is tuned to a photoassociation resonance. Therefore, by detuning to the red side of atomic resonance, the effect of binary collisions can be made to be either very large or very small compared to *γ*_atomic_. The cold atomic gas will have a prominent photoassociation spectrum, and the rate for photodestruction at the peak positions will measure the ground state pair correlation function ∝|*Ψ*_g_(*R*_C_)|^2^ at the Condon points for the transitions. There is a discrete set of Condon points *R*_C_(*υ*), corresponding to the outer turning points for the vibrational levels of the upper molecular state. There are several well-known alkali dimer transitions that have already been studied [7] in much hotter traps than the < 1 μK traps that are now available. The intriguing question remains whether 3-body or collective effects might perturb the binary photoassociation spectrum, causing broadening or shifts in the resonance line profiles. Kagan et al. [45] have suggested the possibility of observing quantum correlations in optical properties of low temperature spin-polarized gases. The photoassociation spectrum of a cold dense atomic gas near BEC might be a very fruitful source of experimental and theoretical investigation of 2- and 3-body interactions in such systems [11].

Certainly the analysis in this paper shows that light can induce significant loss processes, in comparison to that caused by free atom light scattering, due to collisions between atoms in a cold atomic gas near 1 mK or below at densities > 10^13^ cm^−3^. If the density should increase to > 10^15^ cm^−3^, the collisional loss rate will dominate that due to atomic recoil heating, except between photoassociation resonances for large enough red detuning. For the case of the MIT Na trap [3] the blue detuning of the laser used to plug the hole in the magnetic trap was so far off-resonance that any atomic or binary light scattering did not prevent the formation of a condensate. But for much smaller detunings the simple Franck-Condon formulas we have derived here give a way to estimate the binary collision rates for trap loss *via* excited state production. More detailed treatment, including incorporating the actual quasimolecular structure of alkali dimer states, could be readily incorporated into this framework. We have also developed a theory for treating the nonperturbative effect of light, including multiphoton effects and the modification of the ground state scattering length [9]. Careful attention to collective effects also needs to be given for the case of higher densities. In any case, it seems very unlikely that a condensate could be stable for very long in the presence of light tuned within a few hundred GHz of atomic resonance.

## 8. Appendix A. Milne Equation Analysis of Asymptotic Wavefunction

The use of the exact Milne quantum mechanical equations for the phase and amplitude of the wavefunction lets us do an exact analysis of the asymptotic wavefunction. Let the wavefunction be writen in phase-amplitude energy-normalized form:

$$\psi (R,E)={\left(\frac{2\mu }{\pi \hbar^{2}}\right)}^{1/2}\alpha (R,E)\;\sin(\beta (R,E)),$$ (60)

where *α* satisfies the Milne equation [46, 47],

$$\left(\frac{d^{2}}{dR^{2}}+k^{2}(R,E)\right)\alpha (R,E)-\frac{1}{\alpha {\left(R,E\right)}^{3}}=0.$$ (61)

The phase *β* is calculated from *α* by

$$\beta (R,E)=\int \frac{1}{\alpha {\left(R,E\right)}^{2}}dR,$$ (62)

and the local wave vector is

$$k(K,E)=\frac{2\pi }{\lambda (R)}=\sqrt{\frac{2\mu }{\hbar^{2}}(E-V(R))}.$$ (63)

The asymptotic potential is *V*(*R*) = − *C*_6_/R^6^.

In the semiclassical picture, we have the identification of *α* with the WKB amplitude

$$\alpha^{\text{WKB}}(R,E)=\frac{1}{\sqrt{k(R,E)}},$$ (64)

and the above forms are equivalent to the WKB wave-function. The exact solution to the quantum equations [Eqs. (61) and (62)] give an exact quantum wavefunction. Here we will use an asymptotic analysis for the wavefunction at moderately long range.

At long range the amplitude becomes constant, and very large as *E* → 0:

$$\alpha_{\infty }=\frac{1}{\sqrt{k_{\infty }}}=\sqrt{\frac{\lambda_{\infty }}{2\pi }}.$$ (65)

Therefore, we want to calculate the departure of this large, nearly independent of *R*, amplitude from its asymptotic limit. We do this by *approximating* the last term of Eq. (61) by $1/\alpha^{3}\approx k_{\infty }^{2}\alpha$. Then Eq. (61) becomes

$$\frac{d^{2}\alpha }{dR^{2}}+(k^{2}(R)-k_{\infty }^{2})\alpha =0$$ (66)

$$\frac{d^{2}\alpha }{dR^{2}}-\frac{2\mu }{\hbar^{2}}V(R)\alpha =0$$ (67)

$$\frac{d^{2}\alpha }{dR^{2}}=-\left(\frac{2\mu }{\hbar^{2}}C_{6}\right)\frac{1}{R^{6}}\alpha .$$ (68)

Equation (68) can be immediately integrated to give

$$\alpha (R)=\alpha_{\infty }\left(1-\frac{2\mu C_{6}}{20\hbar^{2}R^{4}}\right)$$ (69)

$$=\alpha_{\infty }\left(1-{\left(\frac{R_{B}}{R}\right)}^{4}\right)$$ (70)

where *R*_B_ is defined by Eq. (13). When *R* = 20^1/4^*R*_B_ = 2.1 *R*_B_, the amplitude *α*(*R*) has only changed by 5 % from its asymptotic value *α*. When *R* = *R*_B_, the correction term is unity, and the asymptotic approximation used in setting up the approximate Eq. (66) breaks down. Therefore, the amplitude expression in Eq. (70) can only be applied for *R* ≫ *R*_B_.

Now we can turn our attention to deriving the correction to the asymptotic phase. This is done by substituting the above amplitude, Eq. (70), into the phase [Eq. (62)]. The asymptotic phase at some suitable large value of *R* = *R*_∞_ (a distance well beyond the range of the potential) is *β*_∞_ = *k*_∞_ (*R*_∞_ − *A_s_*), where *A_s_* is the *s*-wave scatter-inglength. The phase function at *R* is calculated by integrating backwards from *R*_∞_,

$$\begin{array}{c} \beta (R)=k_{\infty }(R_{\infty }-A_{s})+\int_{R_{\infty }}^{R}\frac{1}{\alpha {\left(R\right)}^{2}}dR\\ =k_{\infty }(R_{\infty }-A_{s})-k_{\infty }\int_{R}^{R_{\infty }}\frac{dR}{{\left(1-{\left(\frac{R_{B}}{R}\right)}^{4}\right)}^{2}} \end{array}$$ (71)

$$\begin{array}{c} \approx k_{\infty }(R_{\infty }-A_{s})-k_{\infty }\int_{R}^{R_{\infty }}\left(1+2{\left(\frac{R_{B}}{R}\right)}^{4}\right)dR\\ =k_{\infty }\left(R-A_{s}-\frac{2}{3}{\left(\frac{R_{B}}{R}\right)}^{4}R\right). \end{array}$$ (72)

Combining the corrected phase and amplitude expressions, Eqs. (72) and (70), in Eq. (60), we obtain the expression given in Eq. (10) in the text. Note that the normalization stays close to its *asymptotic* value, in spite of the fact that the local *k*(*R*, *E*) can be much larger than *k*_∞_ in that part of the moderately long range region where |*V*_g_| ≫ *E* (see Fig. 2). Thus, the semiclassical local WKB normalization does not apply. Note also that the node of the wavefunction at *R_n_* near *A_s_* for the positive scattering length case is shifted from its value projected from the asymptotic *A*_s_. This is because the phase near *A_s_* has not yet reached its asymptotic limiting value. This shift in the actual node to larger *R* can readily be estimated from Eq. (72):

$$R_{n}-A_{s}-\frac{2}{3}{\left(\frac{R_{B}}{R_{n}}\right)}^{4}R_{n}=0$$ (73)

and

$$R_{n}\approx A_{s}\;\text{implies}\;R_{n}\approx A_{s}\left(1+\frac{2}{3}{\left(\frac{R_{B}}{A_{s}}\right)}^{4}\right).$$ (74)

## 9. Appendix B. Reflection Formula

This derivation of the reflection approximation expression follows Jablonski [48] and the Appendix of Ref. [49]. We begin by writing the ground and excited state wavefunctions in energy-normalized phase-amplitude form

$$\Psi_{g}(R,E)={\left(\frac{2\mu }{\pi \hbar^{2}}\right)}^{1/2}\alpha_{g}(R,E)\;\sin(\beta_{g}(R,E))$$ (75)

$$\Psi_{e}(R,E_{e})={\left(\frac{2\mu }{\pi \hbar^{2}}\right)}^{1/2}\alpha_{e}(R,E_{e})\;\sin(\beta_{e}(R,E_{e})).$$ (76)

where *E_e_* = *E* + *ħ****Δ***_A_. The ground state amplitude and phase are given by Eqs. (70) and (72), whereas the excited state quantities can be taken to be the standard WKB ones:

$$\alpha_{e}(R,E_{e})=\frac{1}{\sqrt{k_{e}(R,E_{e})}}$$ (77)

$$\beta_{e}(R,E_{e})=\int_{R_{t}}^{R}k_{e}(R^{\prime },E_{e})dR^{\prime }+\frac{1}{4}\pi ,$$ (78)

where *R*_t_ is the inner turning point of the repulsive potential. The Franck-Condon factor is

$$F_{\text{eg}}(E,\Delta_{A})=\frac{2\mu }{\pi \hbar^{2}}{\left|I_{\text{eg}}(E,\Delta_{A})\right|}^{2},$$ (79)

where

$$I_{\text{eg}}(E,\Delta_{A})=\int_{0}^{\infty }\alpha_{g}\alpha_{e}\sin\beta_{g}\sin\beta_{e}dR$$ (80)

$$\approx \frac{1}{2}\int_{0}^{\infty }\alpha_{g}\alpha_{e}\;\cos(\beta_{g}-\beta_{e})dR.$$ (81)

In the last line we have made the standard approximation of neglecting the rapidly oscillating phase sum term. The phase difference is expanded in a Taylor series about a point *R*_p_:

$$\phi \left(R,E\right)=\beta_{g}\left(R,E\right)-\beta_{e}\left(R,E_{e}\right)$$ (82)

$$=b_{0}+b_{1}(R-R_{p})+\frac{1}{2}b_{2}{\left(R-R_{p}\right)}^{2}+\ldots ,$$ (83)

where

$$b_{0}=\beta_{g}(R_{p},E)-\beta_{e}(R_{p},E_{e})$$ (84)

$$\approx \beta_{g}(R_{p},E)-\frac{\pi }{4}$$ (85)

$${b_{1}=\frac{d\beta_{g}(R,E)}{dR}|}_{R_{p}}{-\frac{d\beta_{e}(R,E_{e})}{dR}|}_{R_{p}}$$ (86)

$$=\frac{1}{\alpha {\left(R_{p},E\right)}^{2}}-k_{e}(R_{p},E)$$ (87)

$$\approx k_{\infty }-k_{e}(R_{p},E_{e})$$ (88)

$${b_{2}=\frac{d^{2}\beta_{g}(R,E)}{dR^{2}}|}_{R_{p}}{-\frac{d^{2}\beta_{e}(R,E_{e})}{dR^{2}}|}_{R_{p}}$$ (89)

$${\approx -\frac{dk_{e}(R,E_{e})}{dR}|}_{R_{p}}$$ (90)

$$\approx -\frac{\mu D_{C}}{\hbar^{2}k_{e}(R_{p})}.$$ (91)

In the normal semiclassical theory, *R*_p_ is taken to be the Condon point, where *k*_g_ = *k*_e_ so that *b*_1_ = 0. Here we simply take the point where *b*_1_ vanishes. Since the excited state potential is so steep with respect to the effectively flat ground state potential, it is an excellent approximation to take *R*_p_ = *R*_t_ = *R*_C_ to all be equivalent. Then from Eq. (78), *β*_e_(*R*_p_, *E*) = *π*/4. The expression for *b*_2_ follows from neglecting the ground state contribution in comparison to the excited state one.

We thus evaluate

$$\begin{array}{c} I_{\text{eg}}(E)=\alpha_{g}(R_{C},E)\alpha_{e}(R,E_{e})\frac{1}{2}\int_{-\infty }^{\infty }\cos\left(b_{0}+\frac{b_{2}}{2}x^{2}\right)dx\\ =\alpha_{g}(R_{C},E){\left(\frac{\pi \hbar^{2}}{2\mu D_{C}}\right)}^{1/2}\cos\left(\beta_{g}(R_{C},E)-\frac{\pi }{2}\right)\\ ={\left(\frac{\pi \hbar^{2}}{2\mu D_{C}}\right)}^{1/2}\alpha_{g}(R_{C},E)\;\sin(\beta_{g}(R_{C},E)). \end{array}$$ (92)

where we have used $\int_{-\infty }^{\infty }\cos\left(b_{0}+\frac{\left|b_{2}\right|}{2}x^{2}\right)dx$

$$=\frac{2\pi }{\left|b_{2}\right|}\cos\left(b_{0}\pm \frac{\pi }{4}\right).$$ (93)

Using Eqs. (75) and (92) in Eq. (79) gives the desired reflection approximation to the Franck-Condon factor:

$$F_{\text{eg}}(E,\Delta_{A})=\frac{1}{D_{C}}{\left|\Psi_{g}(R_{C},E)\right|}^{2}.$$ (94)
